# ASCIIGenome: a command line genome browser for console terminals

**DOI:** 10.1093/bioinformatics/btx007

**Published:** 2017-01-24

**Authors:** Dario Beraldi

**Affiliations:** Cancer Research UK, Cambridge Institute, Li Ka Shing Centre, University of Cambridge, Cambridge, UK

## Abstract

**Summary:**

Current genome browsers are designed to work via graphical user interfaces (GUIs), which, however intuitive, are not amenable to operate within console terminals and therefore are difficult to streamline or integrate in scripts. To circumvent these limitations, ASCIIGenome runs exclusively via command line interface to display genomic data directly in a terminal window. By following the same philosophy of UNIX tools, ASCIIGenome aims to be easily integrated with the command line, including batch processing of data, and therefore enables an effective exploration of the data.

**Implementation:**

ASCIIGenome is written in Java. Consequently, it is a cross-platform tool and requires minimal or no installation. Some of the common genomic data types are supported and data access on remote ftp servers is possible. Speed and memory footprint are comparable to or better than those of common genome browsers.

**Availability and Implementation:**

Software and source code (MIT License) are available at https://github.com/dariober/ASCIIGenome with detailed documentation at http://asciigenome.readthedocs.io.

**Supplementary information:**

Supplementary data are available at *Bioinformatics* online.

## 1 Introduction

The visualization of next generation sequencing data is a fundamental part of genomics enabling the analyst to assess the quality of the results and generate hypotheses. Accordingly, several programs for browsing genomic data are widely popular amongst the genomics community, for example, IGV (Robinson *et al.*, 2011) or Artemis (Carver *et al.*, 2012) are common choices. Most if not all genome browsers make use of a graphical user interface (GUI), which create an intuitive environment particularly suitable to researchers without programming experience.

For a number of bioinformaticians, however, the command line interface is preferred over the GUI to enable a more efficient data analysis. The command line interface gives finer control over the executed commands and easily streamlines repetitive tasks. In addition, the executed commands can become part of project documentation. Furthermore, genomic data often reside on remote servers and transferring such data locally may be impractical, especially if the analyst only needs a cursory exploration of the data.

ASCIIGenome is a browser designed to run directly from terminal and by means of command line interface. The graphical output is rendered on a terminal window by means of ASCII characters, thus making it suitable to execute on remote servers. Researchers comfortable with the UNIX command line interface should find ASCIIGenome equally familiar. While samtools tview (Li *et al.*, 2009) is designed for terminal window, its functionality is very limited. Some browsers can process data in batch (IGV) or accept command line options (Artemis), but again they rely on the GUI for displaying and interactive browsing. By design, instead, ASCIIGenome has capabilities similar to popular GUI browsers while being fully operated via the command line to visualize data in the terminal window.

## 2 Features

### 2.1 Interface

**Fig. 1.**
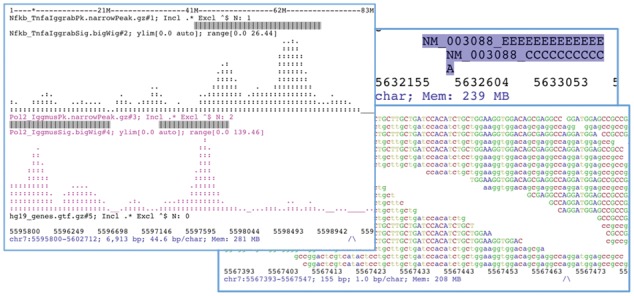
Screenshots from ASCIIGenome. On the left, an example of two ChIP-Seq profiles in bigWig format and two associated bed files showing peak positions. On the right, an example of aligned reads

ASCIIGenome aims at reproducing the behaviour and syntax of the Unix shell familiar to most bioinformaticians. In its interactive mode, ASCIIGenome presents an interface similar to the Unix shell where commands are entered and executed on the fly. As in many terminal shells, previous commands can be browsed by means of the up and down arrow keys and command auto-completion is enabled. In non-interactive (i.e. headless) or batch mode, ASCIIGenome can be used to loop through a file of regions of interest and execute operations on each of them, for example, by zooming out and saving the outputs. Figure 1 shows examples of how genomic data is rendered by ASCIIGenome.

### 2.2 Supported data formats

ASCIIGenome supports several data formats used in genomics (Supplementary Table S1). Most data sources can be read from remote URL addresses such as those available from the UCSC genome browser or Ensembl. Currently, CRAM is a notable format not yet supported although the design of ASCIIGenome does not preclude its inclusion in future releases. Finally, a reference sequence can be loaded from the common fasta format.

### 2.3 Browsing the genome

Since the genomic space is typically large compared with the size of the features of interests, such as ChIP-Seq peaks, ASCIIGenome offers a simple yet powerful set of commands to browse the genome (Supplementary Fig. S1). Besides the typical commands found in most browsers, e.g. moving to a given location or zooming in and out, ASCIIGenome allows to find and filter features based on matching regular expressions or move to the *next* feature on a given file. Currently, 45 commands are available.

Overall, ASCIIGenome makes extensive use of regular expressions to capture features and track names. Consequently, applying the same operation to a subset of tracks can be easily achieved with the appropriate expressions. By combining these and other options with the command line interface, it is quick to explore in detail large genomic regions containing thousands of features. While data are displayed to the user’s terminal in real time, screenshots and subsets of data can be saved in plain text or pdf format.

## 3 Implementation and performance

ASCIIGenome compares favourably to other genome browsers in terms of performance and ease of deployment. By being written entirely in Java, ASCIIGenome should run on most platforms without any need of compilation and installation. In addition, Java offers a good speed of data processing. In terms of memory, ASCIIGenome makes extensive use of indexed files, thus keeping memory usage low since only the portion of data in the current genomic window needs to be loaded. For files already indexed, such as bam, bigWig or tabix indexed files (Li, 2011), ASCIIGenome can directly make use of the indexing without further processing. Tab delimited files without index, such as bed or gff files, are sorted, compressed and indexed to temporary files. Sorting is performed on a temporary sqlite database thus requiring a reasonably small amount of memory and time.

Table 1 shows an informal comparison between ASCIIGenome and IGV. Although the metrics reported here are not directly comparable due to the differences in the interfaces of the two programs, they show how ASCIIGenome can be a fast and lightweight alternative to IGV or similar programs.

**Table 1.** Performance of ASCIIGenome and IGV (Robinson *et al.*, 2011) in loading a gtf and a bam file (see Supplementary Material for details)

	GTF with 2 828 312 records^a^		BAM interval with 2 052 375 reads^b^	
	IGV	ASCIIGenome	IGV	ASCIIGenome
Time	2 m 15 s	2 m 00 s	1 m 15 s	14 s
Memory (GB)	6.48	0.45	6.75	0.73
CPU (%)	808	97	580	184

a This GTF file is neither sorted nor indexed.

b This bam file contained the indicated number of reads on the mitochondrial genome, and this was fully loaded by both IGV and ASCIIGenome.

## 4 Conclusions

ASCIIGenome applies the benefits of the command line interface to the visualization of genomic data thus helping the analysts in efficiently and reproducibly exploring their data. Although the aesthetics of ASCIIGenome output is not as pleasing and feature-rich as that of IGV or similar browsers with GUI, the text organization and colouring retain good expressiveness. In fact, ASCIIGenome aims at complementing GUI-based genome browsers by allowing a fast exploration of the genomic data just like Unix tools such as *less* or *grep* allow a rapid analysis of plain text files.

## Supplementary Material

Supplementary Data
