# Supplementary material for: ASCIIGenome: a command line genome browser for console terminals
Source: Bioinformatics. 2017 Jan 24;33(10):1568–9. doi: 10.1093/bioinformatics/btx007 (PMC5423454; doi:10.1093/bioinformatics/btx007)
Supplement: Supplementary Data [file btx007_supp.docx]

Supplementary data

Comparison between ASCIIGenome and IGV

ASCIIGenome version 0.6.0 was compared to IGV version 2.3.57 in terms of time, memory and CPU usage. Two tasks were assessed: 1) loading of a GTF file gzip compressed, neither sorted nor indexed containing 2,828,312 records and 2) loading of the entire mitochondrial (MT genome from a bam file where the MT genome contains 2,052,375 alignments. The GTF file was deliberately chosen to be unsorted and not indexed because this is how the data are presented when downloaded from Ensembl (see below) or from other data repositories.

Data preparation

The test GTF file was downloaded from Ensembl at <ftp://ftp.ensembl.org/pub/release-75/gtf/homo_sapiens/Homo_sapiens.GRCh37.75.gtf.gz>. The test BAM file was prepared by concatenating 75 times the MT genome extracted from one of the bam files produced by the 1000 genomes project. This code reproduces the test BAM file:

wget <ftp://ftp.ensembl.org/pub/release-75/gtf/homo_sapiens/Homo_sapiens.GRCh37.75.gtf.gz>

samtools view -b HG00096.mapped.ILLUMINA.bwa.GBR.exome.20120522.bam MT > mt.bam

bams=""

for i in {1..75}

do

bams="${bams} mt.bam"

done

samtools merge -f MT.bam $bams ## I.e.: samtools merge -f MT.bam mt.bam mt.bam mt.bam ...

samtools index MT.bam

Testing

Each of the two tasks was executed three times and the performance metrics were collected with the GNU command time. From the output of time, the following metrics were averaged across the three executions: “Percent of CPU”, “Elapsed (wall clock) time”, “Maximum resident set size”.

For ASCIIGenome, the GTF file was loaded as:

/usr/bin/time --verbose ASCIIGenome Homo_sapiens.GRCh37.75.gtf.gz -ni

The BAM file was processed with:

/usr/bin/time --verbose ASCIIGenome -r MT:1-16569 MT.bam -ni

The option –r makes ASCIIGenome move to the given region, i.e. the entire MT genome in our test.

IGV was assessed in a similar way by loading the two files in three executions of each task. The GTF file was loaded with the following batch script

new

genome hg19

load Homo_sapiens.GRCh37.75.gtf.gz

snapshotDirectory .

exit

The batch script to load the BAM file was:

new

genome hg19

load MT.bam

snapshotDirectory .

goto MT:1-16569

exit

IGV was executed by running java with 8 GB of memory (java -Xmx8000m) since the default of 2 GB was insufficient to load the BAM file.

These tests were performed on a server with 32 processing units and running CentOS 6 with the data stored on a network-attached storage server.

Options for interactive browsing

N a v i g a t i o n

goto chrom:[from]-[to]

Go to region `chrom:from-to` or to `chrom:from` or to the start of `chrom`.

INT [INT]

Go to position `INT` or to region `INT INT` on current chromosome.

+ INT [k|m]

Move forward by `INT` bases. Suffix K/M recognized.

- INT [k|m]

Move backwards by INT bases. Suffix K/M recognized.

f [NUM=0.1]

Move forward NUM times the size of the current window, 1/10 by default.

b [NUM=0.1]

Move backward NUM times the size of the current window, 1/10 by default

ff

Move forward by 1/2 of a window. A shortcut for `f 0.5`

bb

Move backward by 1/2 of a window. A shortcut for `b 0.5`

zi [INT = 1]

Zoom in INT times. Each zoom halves the window size.

zo [INT = 1]

Zoom out INT times. Each zoom doubles the window size.

extend [mid|window] [INT left] [INT right]

Extend the current window by `INT` bases left and right.

l

Go to the Left half of the current window.

r

Go to the Right half of the current window.

p

Go to the previous visited position.

n

Go to the next visited position.

next [-back] [-start] [track]

Move to the next feature not overlapping the current coordinates.

F i n d

find [-all] regex [track]

Find the first record in `track` containing `regex`.

seqRegex [-iupac] [-c] [regex]

Find regex in reference sequence and show matches as an additional track.

bookmark [name] | [-rm] | [-print] | [> [file]]

Creates a track to save positions of interest.

D i s p l a y

grep [-i = .*] [-e = ''] [track_regex = .*]...

Similar to grep command, filter for features including or excluding patterns.

featureDisplayMode [-expanded | -collapsed | -oneline] [track_regex = .*]...

Set how annotation features should be displayed.

gap [-on | -off] [track_regex = .*]...

Display features with or without a separating gap.

gffNameAttr [attribute_name = NULL | -na] [track_regex = .*]...

GTF/GFF attribute to set the feature name or `-na` to suppress name.

trackHeight INT [track_regex = .*]...

Set track height to INT lines of text for all tracks matching regexes.

ylim <NUM|min|na> <NUM|min|na> [track_regex = .*]...

Set the y-axis limit for all tracks matched by regexes.

colorTrack color [track_regex = .*]...

Set colour for tracks matched by regex.

hideTitle [-on | -off] [track_regex = .*]...

Set the display of the title line matched by track_regex.

editNames -t <pattern> <replacement> [track_re=.*]...

Edit track names by substituting regex pattern with replacement.

dataCol [index = 4] [track_regex = .*]...

Select data column for bedgraph tracks containing regex.

print [-n INT] [-full] [-off] [track_regex = .*]... [>|>> file]

Print lines for the tracks matched by `track_regex`.

A l i g n m e n t s

rpm [-on | -off] [track_regex = .*]

Set display to reads per million for BAM and TDF files.

samtools [-f INT=0] [-F INT=4] [-q INT=0] [track_re = .*] ...

Apply samtools filters to alignment tracks captured by the list of track regexes.

BSseq [-on | -off] [track_regex = .*]...

Set bisulfite mode for read tracks matched by regex.

G e n e r a l

setGenome fasta|bam|genome

Set genome and reference sequence.

showGenome

Print the genome dictionary with a representation of chromosome sizes.

infoTracks

Print the name of the current tracks along with file name and format.

recentlyOpened

List recently opened files.

addTracks [file or URL]...

Add tracks from local or remote files.

dropTracks [-t] track_regex [track_regex]...

Drop tracks matching any of the listed regexes.

orderTracks [track_regex]...

Reorder tracks according to the list of regexes or sort by name.

posHistory

List the visited positions.

history

List the executed commands.

save [>>] [filename = chrom_start_end.txt']

Save screenshot to file as text or pdf format.

sessionSave filename

Experimental: Save the current settings to file suitable to be reloaded by ASCIIGenome.

q

Quit

h -h

h and -h show this help.

For help on commands: `command -h`, e.g. `ylim -h`

Full documentation at: <http://asciigenome.readthedocs.io/>

**Figure S1.** Options available for interactive browsing. This list can be invoked by entering “h” at the command prompt of ASCIIGenome. Detailed help for each option can be displayed by entering “*command* -h”, e.g. “ylim -h”. See also <http://asciigenome.readthedocs.io/en/latest/commandHelp.html>.

Supported data formats

**Supplementary Table S1.** Data formats supported by ASCIIGenome. All plain text formats (gtf, bed, vcf, *etc*) can be read also as gzip compressed. The file name extension determines how ASCIIGenome interprets the input with default format being bed. See also <http://asciigenome.readthedocs.io/en/latest/supported_io.html#input-file-formats>. For more information on each format see also the Ensembl and UCSC Genome Browser web sites.

| **Format** | **Extension** |
| --- | --- |
| **Annotation** | |
| gtf, gff | .gtf .gff .gff3 |
| bigBed | .bb .bigBed |
| bed | Any (default) |
| **Quantitative data** | |
| bigwig | .bigWig .bw |
| bedGraph | .bedGraph |
| tdf | .tdf |
| **Other** | |
| vcf | .vcf |
| bam | .bam |
